# 167. Efficacy of Investigational Microbiota-Based Live Biotherapeutic RBX2660 in Individuals with Recurrent *Clostridioides difficile* Infection: Data from Five Prospective Clinical Studies

**DOI:** 10.1093/ofid/ofab466.167

**Published:** 2021-12-04

**Authors:** Lindy Bancke, Xin Su

**Affiliations:** 1 Rebiotix, a Ferring Company, Roseville, Minnesota; 2 Rebiotic/Ferring, St Paul, Minnesota

## Abstract

**Background:**

Microbiota-based treatments have shown promise to reduce recurrence, morbidity, and mortality for recurrent *Clostridioides difficile* infections (rCDI), but consistent and reliable clinical efficacy data are needed to support regulatory approvals that broaden patient access. Here we provide cumulative data from 5 prospective clinical studies evaluating RBX2660—a standardized, microbiota-based investigational live biotherapeutic—for reducing rCDI recurrence.

**Methods:**

This analysis included three phase 2 (PUNCH CD, PUNCH CD2, PUNCH CD Open Label) and two phase 3 trials (PUNCH CD3, PUNCH CD3-OLS *ad hoc* analysis). All participants were ≥18 years old with documented rCDI who completed standard-of-care (SOC) oral antibiotic therapy prior to treatment with RBX2660. Depending on the trial, assigned study treatment was 1 or 2 doses of RBX2660 or placebo, with Treatment Success (TS) defined as remaining recurrence-free for 8 weeks after treatment. Treatment responders were monitored for additional recurrence through at least 6 months after receiving the last RBX2660 dose. Treatment non-responders were administered SOC antibiotic treatment and/or additional RBX2660 treatment and monitored for recurrence for 8 weeks after the last received RBX2660 treatment.

**Results:**

Among the 5 trials with a total of 629 participants, RBX2660 consistently reduced the recurrence of rCDI, with TS rates ranging from 50 to 78.9% (Figure 1). Among primary non-responders, additional RBX2660 treatments further reduced recurrence and overall rates of TS ranged from 75.0% to 84.4% (Figure 2). Among CD, CD3, and CD3-OLS, a majority of primary responders remained CDI-free to 6 and up to 24 months with success rates ranging from 74.4% to 92.1%.

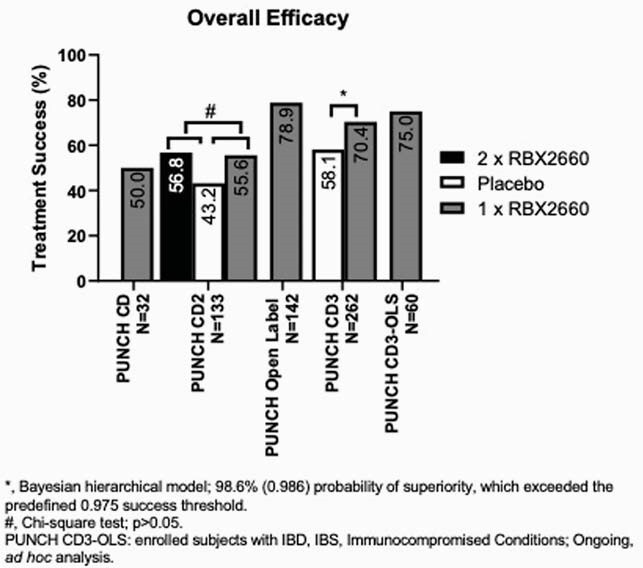

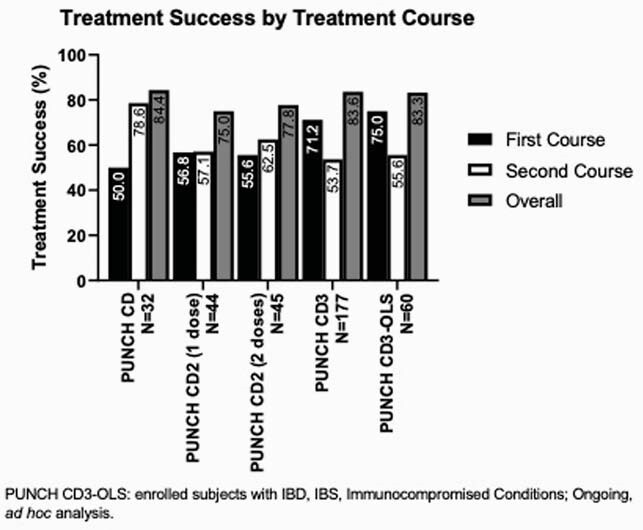

**Conclusion:**

Among 5 trials with consistent investigational product and clinical endpoints, RBX2660 consistently reduced rCDI recurrence, with a majority of treatment responders remaining CDI-free for at least 6 and up to 24 months. Further, initial lack of response to RBX2660 did not preclude clinical benefit of additional RBX2660 treatment. Collectively, these data demonstrate consistency and reliability of the potential benefit of RBX2660 across an entire clinical program.

**Disclosures:**

**Lindy Bancke, PharmD**, **Rebiotix, a Ferring Company** (Employee) **Xin Su, MD**, **Rebiotix/Ferring** (Employee)

